# HIV-1 protective epitope-specific CD8^+^ T cells in HIV-1-exposed seronegative individuals

**DOI:** 10.1016/j.isci.2023.108089

**Published:** 2023-09-29

**Authors:** Takayuki Chikata, Hiroyuki Gatanaga, Hung The Nguyen, Daisuke Mizushima, Yu Zhang, Nozomi Kuse, Shinichi Oka, Masafumi Takiguchi

**Affiliations:** 1 Tokyo Laboratory and Division of International Collaboration Research, Joint Research Center for Human Retrovirus Infection, Kumamoto University, Kumamoto 162-0052, Japan; 2 AIDS Clinical Center, National Center for Global Health and Medicine, Shinjuku, Tokyo 162-8655, Japan

**Keywords:** Immune response, Cell biology

## Abstract

Although previous studies have reported HIV-1-specific T cell responses in HIV-1-exposed seronegative (HESN) individuals, there has been no detailed analysis of these T cells against HIV-1 infection. We investigated HIV-1-specific CD8^+^ T cell responses in 200 Japanese HESN men who have sex with men (MSM). T cell responses to 143 well-characterized HIV-1 epitope peptides were analyzed by intracellular cytokine staining assay consisting of 3-week cultures of PBMCs stimulated with peptides. HLA-B∗51:01-restricted Pol TI8-specific and HLA-A∗02:06-restricted Pol SV9-specific CD8^+^ T cells were identified in two and one individuals, respectively, whereas CD8^+^ T cells specific for other HLA-A∗02:06-restricted or HLA-B∗51:01 epitopes were not present in these individuals. These epitope-specific T cells recognized HIV-1-infected cells. Because these two epitopes were previously reported to be protective in HIV-1-infected individuals, these protective epitope-specific T cells might suppress HIV-1 replication in HESN-MSM individuals. The present study suggests the contribution of protective epitope-specific T cells to protection against HIV-1 infection.

## Highlights

• HIV-1-specific T-cells were identified from 4/200 HESN individuals
• These T cells recognized protective epitopes
• These T cells could recognize HIV-1-infected cells

## Graphical abstract

**Figure:**
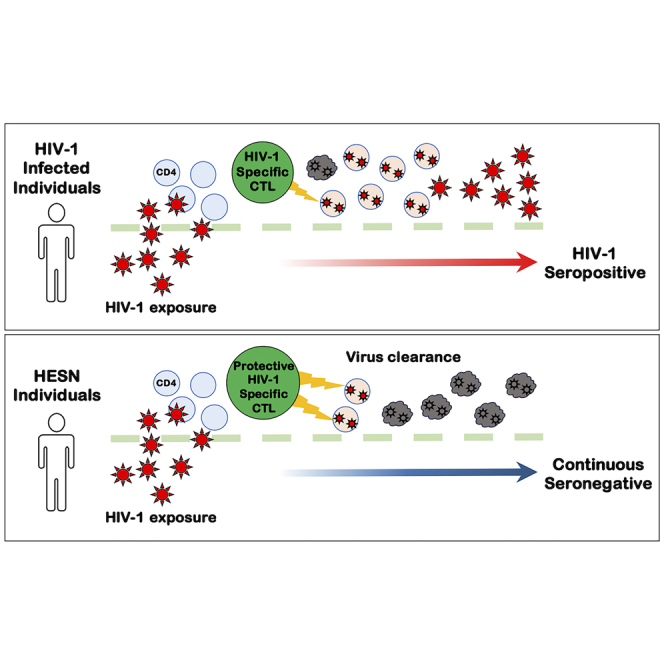

## Introduction

More than 1.5 million people were diagnosed globally as newly infected with HIV-1 in 2021.^1^ Nevertheless, an effective prophylactic AIDS vaccine has not been developed. The clinical trial of an antibody vaccine, RV144 (AIDSVAX B/E and ALVAC-HIV), consisting of priming with a recombinant canarypox vaccine and boosting with an envelope protein, reported a weak effect with a 31% reduction in HIV infection,^2^ although there was an issue with the statistical analysis performed.^3^ The follow-up trial of HVTN702 (ALVAC-HIV and Bivalent Subtype C gp120–MF59) administered to participants in South Africa did not prevent HIV-1 infection during 24 months of follow-up although this vaccine elicited humoral and cellular responses similar to those observed to RV144.^4^ Regardless, a T cell vaccine that elicits HIV-1-specific CD8^+^ T cells was expected to prevent HIV-1 infection because many studies have demonstrated that HIV-1-specific cytotoxic T lymphocytes (CTLs) have an important role in suppressing HIV-1 replication in those with acute or chronic HIV-1 infection.^5^^,^^6^^,^^7^^,^^8^^,^^9^ However, clinical trials of T cell vaccines were not successful, even when HIV-1-specific T cells were elicited.^10^^,^^11^^,^^12^^,^^13^^,^^14^^,^^15^ To date, no successful trials of an AIDS vaccine have been reported.

The relative risk of HIV-1 infection correlates directly with the number of exposures to the virus. However, some individuals remain uninfected despite unprotected encounters with HIV-1-infected individuals in cohorts of HIV-1 seronegative discordant couples, commercial sex workers with unprotected sexual contact, and men who have sex with men (MSM) within regions endemic for HIV-1.^16^^,^^17^^,^^18^^,^^19^ These HIV-1-exposed seronegative (HESN) individuals might reflect acquired or natural protection against the virus. It is well-known that some HESN individuals have an inherited genetic mutation, specifically a 32-bp deletion (Δ32) of the CCR5 co-receptor for HIV-1.^20^^,^^21^ Alternatively, several groups have hypothesized that T cell immunity or innate immunity may protect the establishment of HIV-1 infection in HESN individuals. Indeed, HIV-1-specific T cell responses have been detected in some HESN individuals.^22^^,^^23^^,^^24^^,^^25^^,^^26^^,^^27^^,^^28^^,^^29^^,^^30^^,^^31^^,^^32^^,^^33^^,^^34^^,^^35^^,^^36^^,^^37^ However, whether HIV-1-specific T cell responses are a definitive cause of the protection remains unknown.

A recent study of 412 HESN individuals and 84 eventually seroconverted individuals reported that the number of Integrase/Vif/Nef-specific T cells measured by an IFN-γ ELISpot assay was higher in persistently HESN individuals than in pre-infected samples from seroconverted individuals, suggesting that these T cells contribute to reducing the infection risk.^33^ However, the HIV-1 epitopes recognized by these T cells were not identified and whether HIV-1-infected cells are recognized by these T cells was not analyzed in this study.^33^ Although several studies using HIV-1 peptides have reported T cell responses from samples taken from a cohort of HESN individuals, these HIV-1-specific T cell responses were low in magnitude and breadth,^28^^,^^36^^,^^38^^,^^39^^,^^40^^,^^41^ and transiently detectable.^42^^,^^43^ Furthermore, few studies have successfully established robust T cell lines specific to HIV-1 peptide.^30^^,^^31^^,^^43^^,^^44^ Thus, the features and functions of HIV-1-specific T cells identified in HESN individuals are only partially understood.

Because the number of HIV-1-specific T cells in HESN individuals is presumed to be very small, it is very difficult to efficiently expand and detect these T cells *in vitro*. Stimulating PBMCs with well-characterized epitope peptides instead of overlapping HIV-1 peptide pools followed by culturing might allow the expansion of HIV-1-specific T cells. Therefore, we stimulated PBMCs from 200 Japanese HESN-MSM individuals with large numbers of well-characterized HIV-1 epitope peptides and cultured them for 3 weeks to identify HIV-1-specific CD8^+^ T cells. We also analyzed the epitope specificity of these CD8^+^ T cells and their ability to recognize HIV-1-infected cells.

## Results

### Detection of HIV-1-specific T cells in HESN-MSM individuals

**Figure 1:**
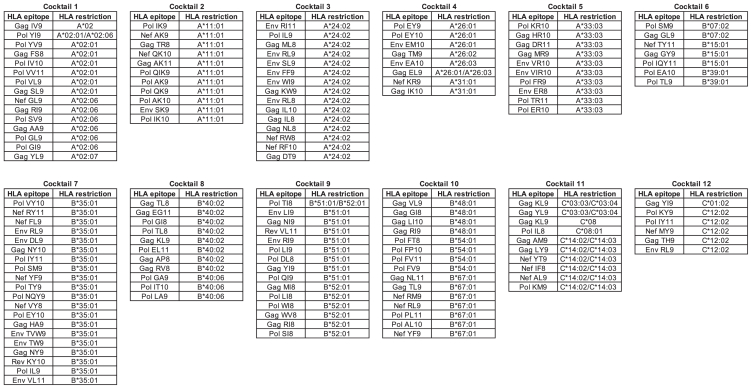
A list of HIV-1 epitope peptides in 12 peptide cocktails.

**Figure 2:**
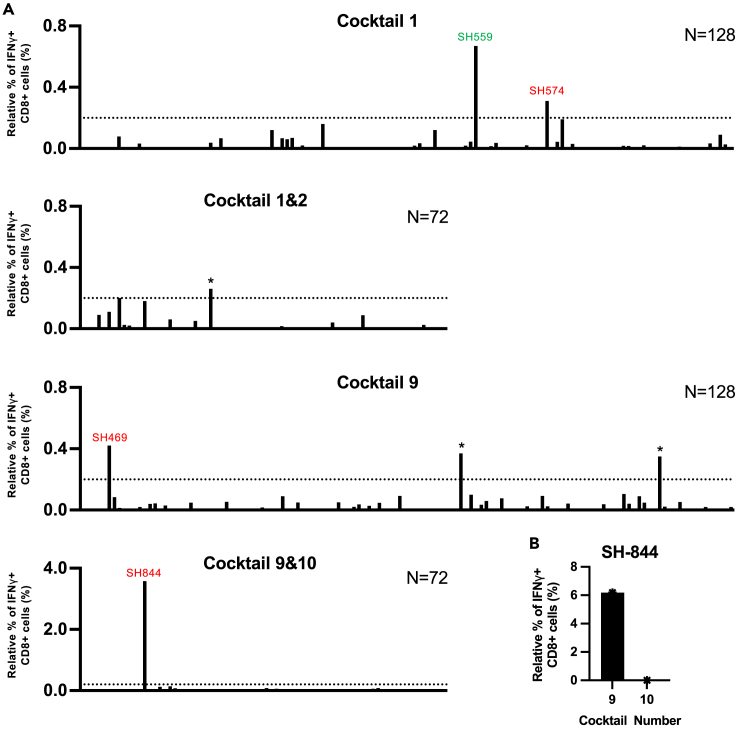
Identification of CD8^+^ T cells specific for HIV-1 peptide cocktails in HESN-MSM individuals (A) T cell responses to each peptide cocktail in 128 individuals or to two peptide cocktails in 72 individuals were analyzed by ICS assay. ∗>0.2% relative frequency of IFN-γ^+^CD8^+^ T cells but evaluated as a non-HIV-1-specific response due to the high background response. All IFN-γ^+^CD8^+^ T cells with a relative frequency of 0.1%–0.2% had high background. (B) Cultured T cells stimulated with Cocktails 9 and 10 in SH-844 were re-analyzed for .221-B5101 cells prepulsed with Cocktail 9 or Cocktail 10.

We analyzed PBMCs from 200 Japanese HESN-MSM individuals to identify HIV-1 epitope-specific CD8^+^ T cells. To induce HIV-1-specific T cells by culturing PBMCs, we used 143 well-defined CD8^+^ T cell epitopes restricted by 28 HLA alleles which cover most Japanese individuals (Figure 1). These epitopes were protective, immunodominant, or subdominant and well-characterized in HIV-1 infected Japanese cohorts (Table S1). We generated 12 peptide-cocktails including 7–20 epitopes peptides and then 3 × 10^5^ or 6 × 10^5^ PBMCs were stimulated with each peptide cocktail (128 individuals) or with two cocktails (72 individuals). After 3 weeks of culture, we analyzed the responses of the cultured T cells to peptide cocktail pulsed 721.221 or C1R transfectants expressing a corresponding HLA allele by performing an IFN-γ intracellular cytokine staining (ICS) assay. We found positive responses to Cocktail 9 in 1 individual (SH-469), Cocktail 1 in 2 individuals (SH-574 and SH-559), and Cocktails 9/10 in 1 individual (SH-844), but no positive response to any peptide cocktails in the other 196 individuals (Figure 2A). Further analysis demonstrated a positive response to Cocktail 9 but not to Cocktail 10 in SH-844 (Figure 2B). These results indicate that CD8^+^ T cells specific for epitope(s) in Cocktail 1 or Cocktail 9 were elicited in these four individuals.

Unfortunately, we could not expand T cells specific for Cocktail 1 in SH-559 for further analysis such as epitope identification. Therefore, we sought to identify epitope-specific T cell responses in three individuals (SH-469, SH-574, and SH-844). We performed HLA typing and identified their HLA alleles: HLA-A∗02:01/31:01, HLA-B∗51:01/-, and HLA-C∗14:02/15:02 in SH-469; HLA-A∗02:06/24:02, HLA-B∗07:02/48:01, and HLA-C∗07:02/08:01 in SH-574; and HLA-A∗02:07/31:01, HLA-B∗46:01/51:01, and HLA-C∗01:02/14:02 in SH-844. Cocktail 1 includes HLA-A∗02:01, -A∗02:06, and -A∗02:07 epitopes and Cocktail 9 includes HLA-B∗51:01 and HLA-B∗52:01 epitopes. These findings suggest that SH-469 and SH-844 have HLA-B∗51:01-restricted T cells whereas SH-574 has HLA-A∗02:06-restricted T cells.

We followed SH-469, SH-559, and SH-844 for 31, 30, and 10 months, respectively, after blood collection and found they were HIV-1 seronegative and p24 antigen negative during these periods. SH-574 dropped out of this cohort after blood collection for this study. We therefore tested HIV-1 RNA in a plasma sample from SH-574 by PCR and found they were HIV-1 RNA-negative. These findings suggest that these four individuals had not been infected with HIV-1, at least when their PBMCs were collected for the current study. These three responders were not infected with HIV-1 between 10 and 31 months, whereas 5 of 196 non-responders were infected with HIV-1 between 10 and 33 months. These findings suggest that these HIV-1 protective epitope-specific T cells might contribute to preventing HIV-1 infection.

### Identification of HIV-1 epitope-specific CD8^+^ T cells in HESN-MSM individuals

**Figure 3:**
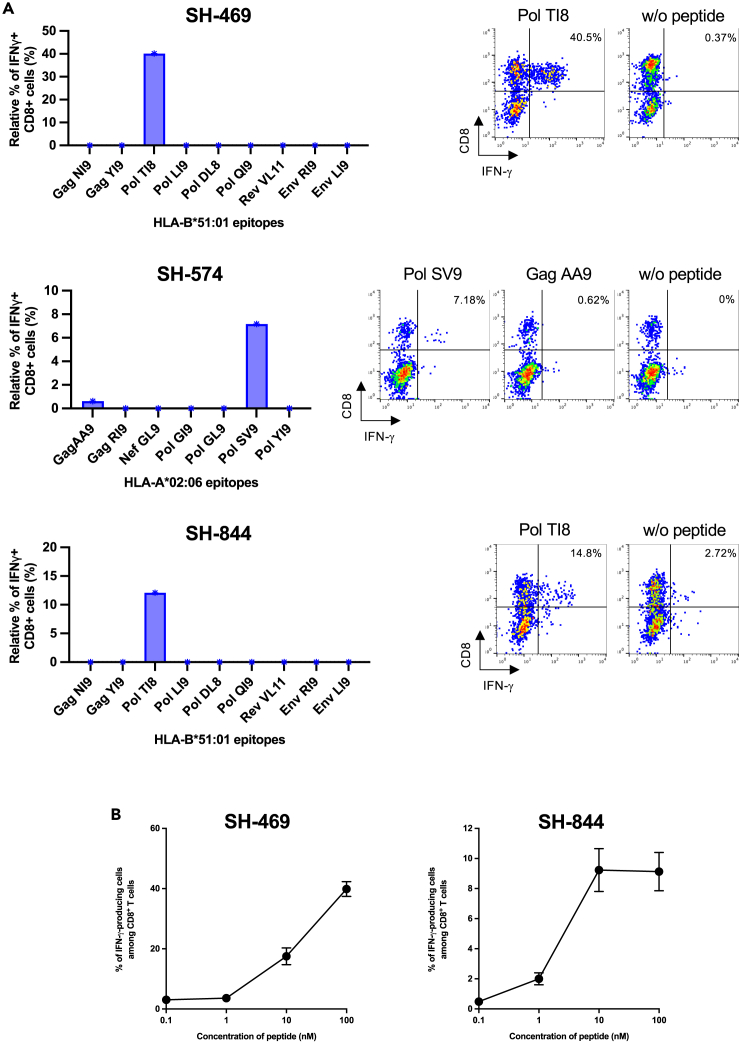
Identification of a single epitope recognized by CD8^+^ T cells specific for HIV-1 peptide cocktails (A) HLA-B∗51:01-restricted and HLA-A∗02:06-restricted CD8^+^ T cell responses to single epitope peptides. Responses of bulk T cells derived from SH-469 and SH-844 to .221-B5101 cells prepulsed with single epitope peptides in Cocktail 9 or those from SH-574 to 221-A0206 prepulsed with single epitope peptides in Cocktail 1 at a concentration of 1 μM were analyzed by ICS assay. Relative frequency of IFN-γ^+^CD8^+^ T cells (left) and frequency of IFN-γ^+^CD8^+^ T cells by flow cytometry analysis (right). (B) Sensitivity of HLA-B∗51:01-restricted Pol TI8-specific CD8^+^ T cells. The responses of HLA-B∗51:01-restricted Pol TI8-specific T cells to .221- B5101 cells pre-pulsed with Pol TI8 peptides at concentrations between 0.1 and 100 nM were analyzed by ICS assay.

Next, we identified epitopes recognized by these T cells. We used single HLA-A∗02:06-restricted or HLA-B∗51:01-restricted epitope peptides in Cocktail 1 or Cocktail 9 and a transfectant expressing HLA-A∗02:06 or HLA-B∗51:01. CD8^+^ T cells from SH-469 and SH-844 recognized the HLA-B∗51:01-restricted epitope Pol TI8, and those from SH-574 strongly recognized the HLA-A∗02:06-restricted epitope Pol SV9 and faintly recognized the HLA-A∗02:06-restricted epitope GagAA9 (Figure 3A left). The CD8^+^ T cell response to GagAA9 was at the background level (Figure 3A right). These results confirmed the HLA-A∗02:06-restricted response to Cocktail 1 and HLA-B∗51:01-restricted response to Cocktail 9 and clearly demonstrated the existence of Pol SV9-specific and Pol TI8-specific T cell responses in these individuals. In addition, T cells specific for other HLA-A∗02:06-restricted or HLA-B∗51:01-restricted epitopes were not elicited in these individuals (Figure 3A left). HLA-B∗51:01-restricted Pol TI8 specific CD8^+^ T cells established from the two individuals recognized the peptides dose-dependently, and even at a concentration of 10 nM (Figure 3B), suggesting they had a relatively strong recognition of the epitope peptides. Interestingly, HLA-B∗51:01-restricted Pol TI8 and HLA-A∗02:06-restricted Pol SV9 were reported to be protective HIV-1 epitopes in HIV-1-infected Japanese individuals.^45^^,^^46^^,^^47^ Thus, only protective HIV-1 epitope-specific CD8^+^ T cells were elicited in HESN-MSM Japanese individuals.

### Recognition of HIV-1-infected cells by HIV-1-specific T cells derived from HESN-MSM individuals

**Figure 4:**
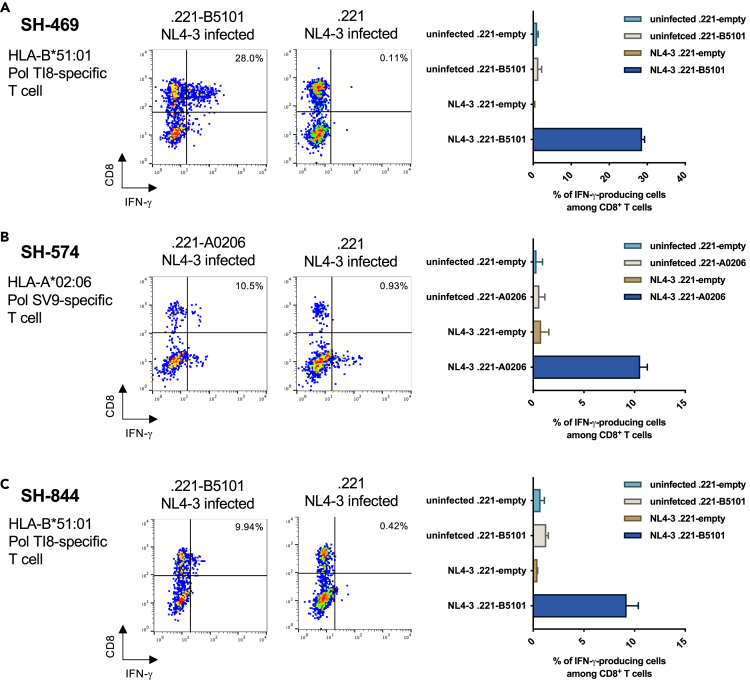
Recognition of NL4-3-infected cells by Pol TI8-specific and Pol SV9-specific CD8^+^ T cells (A–C) The responses of Pol TI8-specific CD8^+^ T cells from SH-469 or SH-844 to NL4-3-infected .221-B5101 cells, NL4-3-infected .221 cells, uninfected .221-B5101 cells, or uninfected .221 cells (A and C) and those of Pol SV9-specific CD8^+^ T cells to NL4-3-infected .221-A0206, NL4-3-infected .221 cells, uninfected .221-A0206 cells, or uninfected .221 cells (B) were analyzed by ICS assay. Representative results of flow cytometric analysis (left) and analysis of triplicate samples (right) are presented. Data are expressed as mean ± SD. Frequency of IFN-γ^+^CD8^+^ T cells is shown. The frequencies of p24 antigen-positive cells among NL4-3-infected .221-B5101 cells or NL4-3-infected .221-A0206 cells and NL4-3-infected .221 cells in each analysis are as follows: A: .221-B5101(30.8%) and .221 cells (63.9%) for the analysis of SH-469-derived Pol TI8-specific CD8^+^ T cells, B: .221-A0206 (46.3%) and .221 cells (21.4%) for the analysis of SH-574-derived Pol SV9-specific CD8^+^ T cells, and C: .221-B5101 (59.3%) and .221 cells (47.0%) for the analysis of SH-844-derived Pol TI8-specific CD8^+^ T cells.

HLA-A∗02:06-restricted Pol SV9-specific and HLA-B∗51:01-restricted Pol TI8-specific CD8^+^ T cells established from HIV-1-infected individuals recognized HIV-1-infected cells and/or had the ability to suppress HIV-1 replication.^45^^,^^46^^,^^47^ Therefore, we assumed T cells from HESN individuals would also recognize HIV-1-infected cells. To clarify whether HIV-1-specific T cells from HESN-MSM individuals recognize HIV-1-infected cells, we analyzed the recognition of Pol TI8- and Pol SV9-specific T cells established from SH-469, SH-844, and SH-574, for NL4-3-infected .221-B5101 and NL4-3-infected .221-A0206 cells, respectively. Pol TI8-specific CD8^+^ T cells recognized NL4-3-infected .221-B5101 cells but not uninfected .221-B5101 cells, and Pol SV9-specific CD8^+^ T cells recognized NL4-3-infected .221-A0206 cells but not uninfected .221-A0206 cells (Figure 4). These results indicate that CD8^+^ T cells specific for these two epitopes from HESN individuals recognized HIV-1-infected cells. Previous studies demonstrated that CD8^+^ T cells specific to these two epitopes from HIV-1-infected individuals suppressed HIV-1 replication and killed HIV-1-infected cells,^45^^,^^46^ implying that T cells specific to the same epitopes as those in HESN individuals can kill HIV-1-infected cells.

## Discussion

Previous studies have shown that HIV-1-specific T cells are elicited in HESN individuals,^22^^,^^23^^,^^24^^,^^25^^,^^26^^,^^27^^,^^28^^,^^29^^,^^30^^,^^31^^,^^32^^,^^33^^,^^34^^,^^36^^,^^48^ suggesting such T cells might protect against HIV-1 infection. However, because most of these studies analyzed only T cell responses to overlapping HIV-1 peptides and/or pools of these peptides in HESN individuals, the epitopes recognized by these T cells and the characterization of HIV-1-specific T cells in HESN individuals remain unclear. In the current study, we aimed to directly identify CD8^+^ T cells specific for HIV-1 epitopes in HESN individuals by using large numbers of well-characterized epitope peptides. Out of 200 HESN-MSM individuals analyzed, we identified CD8^+^ T cells specific for HLA-B∗51:01-restricted Pol TI8 or HLA-A∗02:06-restricted Pol SV9 in 3 individuals. A previous study of a sex-worker cohort in Gambia detected HIV-1-specific T cells that recognized HLA-B∗35:01-restricted Nef VY8, Pol HY9, Gag PY9, and Gag NY9 epitopes from seronegative donors,^31^ and the same group subsequently established T cells specific for HLA-A2, -A33, -A∗68:02, -B14, -B35, -B49, and -B7-restricted epitopes in a sex-worker cohort in Kenya.^24^^,^^43^ Another study of an MSM cohort in the USA revealed the induction of Gag KR9-specific CTLs from seronegative donors and confirmed their HLA-A∗03:01-restriction.^30^ Thus, only three previous studies have identified the epitopes and HLA restriction of HIV-1-specific T cell responses in HESN individuals. Here, we report additional cases of CD8^+^ T cells specific for two HIV-1 epitopes in HESN individuals. Because the ability of these previously-reported HIV-1-specific T cells to recognize HIV-1 virus-infected cells was not analyzed, whether HIV-1-specific CD8^+^ T cells in HESN individuals can recognize HIV-1-infected cells remains unknown. In the current study, we clearly demonstrated that Pol TI8-specific and Pol SV9-specific CD8^+^ T cells established from HESN-MSM individuals recognized HIV-1 NL4-3-infected cells.

Different methods were used to identify HIV-1-specific T cells in HESN individuals. Most previous studies used ELISpot assays using pooled peptides covering each HIV-1 protein. Although the ELISpot assay is useful for analyzing many samples, it detects some non-specific responses and is limited to the detection of responses of a small number of HIV-1-specific T cells. >0–50 HIV-1–specific SFU/10^6^ PBMCs were defined as positive responses in most previous studies of HESN individuals,^24^^,^^25^^,^^28^^,^^32^^,^^33^^,^^35^^,^^36^^,^^37^^,^^39^^,^^40^^,^^41^^,^^43^^,^^44^^,^^48^ whereas >100 HIV-1–specific SFU/10^6^ PBMC were accepted as positive response in recent studies of HIV-1-infected individuals. Therefore, positive responses to HIV-1 peptides in previous HESN studies may include negative responses. A previous study using an ICS assay of *ex vivo* PBMC samples from HESN individuals with a 15-mer overlapping HIV-1 peptide pool demonstrated that the average frequency of IFN-γ^+^ T cells in CD3^+^ T cells was less than 1%,^33^ indicating that HIV-1-specific T cell responses were very weak. HIV-1-specific T cells can be detected 10–14 days after the culture of PBMCs from some but not all HIV-1-infected individuals by ICS assay. From these findings, we speculated that it would be difficult to detect HIV-1-specific T cells in a 2-week culture of PBMCs from HESN individuals. Therefore, we used the ICS assay to analyze a 3-week culture of PBMCs from HESN-MSM individuals.

We only detected HIV-1-specific CD8^+^ T cells in 4/200 individuals (2%). In contrast, previous studies of sex-worker cohorts in Gambia and Kenya, and an MSM cohort in the USA detected HIV-1 specific CTLs in 50%, 48%, and 33% of donors tested, respectively, although the number of individuals tested was small (n = 20, 21, 12, respectively).^24^^,^^30^^,^^31^ The difference in the frequency of HIV-1-specific T cells between our cohort and others may be related to the risk rate of infection in HESN individuals. HIV prevalence among female sex workers was 1.8% in high-income countries and 11.8% in developing countries.^49^ Indeed, 35% of female sex-workers in Gambian towns were infected with HIV.^31^ The incidence of HIV among MSM in lower middle-income countries was >5%/year, whereas it was around 2%/year (USA was 0.7%–6.7%/year) in high-income countries.^50^ Although we recruited an MSM population as a high-risk cohort in Japan, the risk of HIV infection in MSM individuals in Japan is relatively low (HIV-1 incidence rate: 3.4%/year, estimated prevalence: 3.04%) compared with endemic areas in other countries.^51^ Thus, the lower frequency of HIV-1 specific T cells in Japanese HESN-MSM individuals might be related to their lower risk of exposure to HIV-1.

Although we used 143 well-characterized epitope peptides in our study, we found HIV-1-specific CD8^+^ T cells in four individuals and identified HLA-B∗51:01-restricted Pol TI8-specific and HLA-A∗02:06-restricted Pol SV9-specific T cells in three individuals. HLA-B∗51:01-restricted Pol TI8 and HLA-A∗02:06-restricted Pol SV9 were previously reported to be immunodominant epitopes but why T cells specific for other immunodominant epitopes were not elicited in these individuals is unclear. The protein level of HLA alleles on HIV-1 infected cells is a critical factor for T cell priming.^52^ Because the expressions of HLA-A and HLA-B were higher than that of HLA-C,^53^^,^^54^^,^^55^ even in HIV-1-infected cells, HLA-A- or HLA-B-restricted T cells may be primed more immediately and effectively after exposure to HIV-1 than HLA-C-restricted T cells. Indeed, the number of reported HLA-C-restricted HIV-1 epitopes is much smaller than that of HLA-A-restricted or HLA-C-restricted epitopes (LANL-HSD: www.hiv.lanl.gov). In addition, a recent study of HIV-1-specific T cell priming from naive T cells demonstrated that HLA-B-restricted T cells were primed from naive T cells whereas HLA-C-restricted ones were not in the same individuals.^55^ Other studies demonstrated that the peptide binding affinity was positively associated with the frequency of T cell responses in HIV-1-infected individuals^56^ and that peptide stability may mediate T cell immunodominance.^57^ These studies suggest that HIV-1-specific T cells are preferentially induced after exposure to HIV-1 in HESN individuals if the binding affinity and/or stability of the epitope peptide is high. Indeed, HLA-B∗51:01-restricted Pol TI8 was one of two peptides that had the highest affinity among HLA-B∗51:01-restricted epitopes.^47^ The 143 epitopes included immunodominant epitopes such as HLA-A∗02-restricted Gag AA9, Gag YL9, Pol IV10, Pol VV11, A∗24:02-restricted Gag KW9, Env RI11, Env RL9, Env FF9, Env WI9, Env RL8, Nef RW8, Nef RF10, A∗31:01-restricted Gag IK10, Nef KR9, HLA-B∗48:01-restricted Gag GI 8, Gag RI9, Gag VL9 and HLA-B∗51:01-restricted Gag YI9, Gag NI9, Pol LI9, Rev VL11, Env RI9, Pol DL8, and Pol QI9 (Table S1). However, T cells specific for these epitopes were not detected in our HESN-MSM cohort and even in the three individuals in whom HIV-1-specific T cells were present. This suggests that other factors may also regulate the induction of T cells.

HLA-B∗51:01-restricted Pol TI8 and HLA-A∗02:06-restricted Pol SV9 are protective HIV-1 epitopes as previously reported, and T cell responses to these epitopes showed a strong correlation with a better clinical outcome in HIV-1-infected Japanese individuals.^58^^,^^59^ Pol TI8-specific CD8^+^ T cells had a strong ability to suppress HIV-1 replication in HIV-1-infected HLA-B∗51:01^+^ Japanese hemophiliacs^60^ although escape mutations have accumulated in this epitope.^61^ Interestingly, a recent analysis demonstrated that Pol TI8-specific CD8^+^ T cells still have a strong ability to suppress HIV-1 replication in HIV-1-infected Japanese individuals,^59^ suggesting that Pol TI8-specific CD8^+^ T cells are elicited in individuals infected with the wild-type virus or a mutant virus with an TI8-8V epitope that is cross-recognized by Pol TI8-specific CD8^+^ T cells.^46^ Another study showed the early presentation of protective epitopes correlated with rapid CD8^+^ T cell activation and the clearance of virus-infected cells.^62^ These findings suggest that protective HIV-1 epitope-specific T cells might contribute to preventing HIV-1 infection in HESN individuals and that these epitopes might be target antigens for HIV-1 prophylactic vaccines. Whether HLA-B∗51:01-restricted Pol TI8 and HLA-A∗02:06-restricted Pol SV9 epitopes are presented earlier and more effectively on HIV-1 infected cells than other epitopes and whether these T cells have a TCR with a higher affinity than other T cells is unknown. Further analyses of these protective epitopes and HIV-1-specific T cells in HESN individuals are necessary to clarify the role of HIV-1-specific T cells in HIV-1 infection. Three responders to protective epitopes were not infected with HIV-1 between 10 and 31 months, whereas 5 of 196 non-responders were infected with HIV-1 between 10 and 33 months. However, it was difficult to statistically analyze the difference in the frequency of HIV-1 infection between the two groups because half of these individuals received pre-exposure prophylaxis (PrEP) or had dropped out of this cohort within 1 year. Further analysis using a large cohort will clarify the role of protective epitope-specific T cells for the prevention of HIV-1 infection.

When considering the development of AIDS vaccines and curative treatments, it is important to clarify the role of protective epitope-specific T cells at mucosal sites and peripheral lymph nodes in HESN individuals and HIV-1-infected individuals. Although it is difficult to collect samples from the mucosal sites and peripheral lymph nodes of HESN individuals in whom protective epitope-specific T cells were detected in PBMC, the analysis of these T cells at these sites in HESN and HIV-1-infected individuals will contribute to the development of AIDS vaccines and curative treatments for HIV-1-infected individuals under combined antiretroviral therapy.

We demonstrated HIV-1-specific CD8^+^ T cells in four HESN-MSM individuals that were HIV-1 seronegative and p24 antigen negative when their blood samples were collected for the current study. In addition, three of the four individuals were followed for 10–31 months after the sampling time and laboratory tests showed that HIV-1 antibodies and p24 antigen were not present during this period, and HIV-1 RNA was not present in the fourth individual. These results strongly suggest that these individuals were not infected with HIV-1, at least when their samples were analyzed. In summary, HIV-1 protective epitope-specific CD8^+^ T cells were elicited in HESN-MSM individuals who are frequently exposed to HIV-1 but not infected with HIV-1. From these findings, it is expected that protective epitope-specific CD8^+^ T cells might effectively suppress HIV-1 replication in HIV-1-infected individuals and contribute to protecting against HIV-1 infection.

### Limitations of the study

In this study, we demonstrated HIV-1-specific T cells in PBMC samples from HESN individuals in a cohort of Japanese MSM. However, we could not perform further analysis of HIV-1-specific T cells (e.g., the phenotype of T cells, the maintenance of T cells) in HESN individuals who were detected HIV-1-specific T cells. We collected very limited blood samples (6 mL) from HESN individuals for this study, and could not collect longitudinal blood samples because approximately half of individuals received pre-exposure prophylaxis (PrEP) within a year or some individuals dropped out of the cohort.

## STAR★Methods

### Key resources table

**Table:** 

REAGENT or RESOURCE	SOURCE	IDENTIFIER
**Antibodies**		

Pacific Blue-conjugated anti-CD3 antibody	Biolegend	Cat# 300330; RRID: AB_10551436
APC-conjugated anti-CD8 antibody	Biolegend	Cat# 300912; RRID: AB_314116
FITC-conjugated anti-CD19 antibody	Biolegend	Cat# 302206; RRID: AB_314236
PE-conjugated anti-IFNg antibody	Biolegend	Cat# 502509; RRID: AB_315234
HIV-1 core antigen-FITC (Clone KC57)	Beckman Coulter	Cat# 6604665; RRID: AB_1575987
Fixable Near IR	Thermo Fisher Scientific	Cat# L10119

**Bacterial and virus strains**		

HIV-1 (NL4-3)	Adachi et al.^82^	N/A

**Biological samples**		

Human blood (HIV-negative MSM individuals)	National Center for Global Health and Medicine, Japan	N/A

**Chemicals, peptides, and recombinant proteins**		

Ficoll Paque Plus	Cytiva	Cat# 17-1440-03
RPMI1640 medium	Gibco, Thermo Fisher Scientific	Cat# C11875500CP
Fetal Bovine Serum (FBS)	Sigma-Aldrich	Cat# 172012
Hygromycin B, Streptomyces sp.	Sigma-Aldrich	Cat# 400051
G 418 disulfate salt	Sigma-Aldrich	Cat# A1720
Recombinant Human IL-2	PeproTeck	Cat# 200-02
MEM Non-Essential Amino Acids Solution (100X)	Gibco, Thermo Fisher Scientific	Cat# 11140050
Sodium Pyruvate (100 mM)	Gibco, Thermo Fisher Scientific	Cat# 11360070
Saponin from quillaja bark	Sigma-Aldrich	Cat# S7900
Brefeldin A	Sigma-Aldrich	Cat# B7651
BD CellFIX	BD Biosciences	Cat# 340181
HIV-1 epitope peptide (all peptide in this study), see Table S1	Summarized in Table S1	N/A

**Experimental models: Cell lines**		

Human: HLA-A∗02:01-expressing C1R cells	Karaki et al.^78^	N/A
Human: HLA-A∗02:06-expressing 721.221 cells (.221-A0206)	Watanabe et al.^65^	N/A
Human: HLA-A∗02:07-expressing C1R cells	Watanabe et al.^73^	N/A
Human: HLA-A∗11:01-expressing 721.221 cells	Fukada et al.^79^	N/A
Human: HLA-A∗24:02-expressing 721.221 cells	Koizumi et al.^74^	N/A
Human: HLA-A∗26:01-expressing C1R cells	Ogawa et al.^69^	N/A
Human: HLA-A∗26:02-expressing C1R cells	Kawashima et al.^68^	N/A
Human: HLA-A∗26:03-expressing C1R cells	Kawashima et al.^68^	N/A
Human: HLA-A∗31:01-expressing C1R cells	Borghan et al.^70^	N/A
Human: HLA-A∗33:03-expressing 721.221 cells	Hossain et al.^72^	N/A
Human: HLA-B∗07:02-expressing 721.221 cells	Kloverpris et al.^80^	N/A
Human: HLA-B∗15:01-expressing C1R cells	Falk et al.^67^	N/A
Human: HLA-B∗35:01-expressing 721.221 cells	Tomiyama et al.^64^	N/A
Human: HLA-B∗39:01-expressing C1R cells	Ogawa et al.^69^	N/A
Human: HLA-B∗40:02-expressing 721.221 cells	Watanabe et al.^73^	N/A
Human: HLA-B∗40:06-expressing 721.221 cells	Murakoshi et al.^58^	N/A
Human: HLA-B∗48:01-expressing 721.221 cells	Murakoshi et al.^71^	N/A
Human: HLA-B∗51:01-expressing 721.221 cells (.221-B5101)	Tomiyama et al.^64^	N/A
Human: HLA-B∗52:01-expressing 721.221 cells	Yagita et al.^75^	N/A
Human: HLA-B∗54:01-expressing 721.221 cells	Hashimoto et al.^66^	N/A
Human: HLA-B∗67:01-expressing 721.221 cells	Murakoshi et al.^58^	N/A
Human: HLA-C∗01:02-expressing 721.221 cells	Watanabe et al.^65^	N/A
Human: HLA-C∗03:03-expressing 721.221 cells	Murakoshi et al.^81^	N/A
Human: HLA-C∗03:04-expressing 721.221 cells	Murakoshi et al.^58^	N/A
Human: HLA-C∗08:01-expressing 721.221 cells	Murakoshi et al.^58^	N/A
Human: HLA-C∗12:02-expressing 721.221 cells	Honda et al.^76^	N/A
Human: HLA-C∗14:02-expressing 721.221 cells	Lin et al.^77^	N/A
Human: HLA-C∗14:03-expressing 721.221 cells	Lin et al.^77^	N/A

**Software and algorithms**		

FlowJo v10.8.1 software	Tree Star, Inc.	https://www.flowjo.com
Prism 9.5.1 software	GraphPad Software	https://www.graphpad.com/features

**Other**		

Peptide synthesizing service	Scrum Inc.	https://www.scrum-net.co.jp/eng
HLA genotyping service	HLA Laboratory	https://hla.or.jp

### Resource availability

#### Lead contact

Further information and requests for resources and reagents should be directed to and will be fulfilled by the Lead Contact, Takayuki Chikata (tchikata@kumamoto-u.ac.jp).

#### Materials availability

This study did not generate new unique reagents.

### Experimental model and study participant details

#### Human subjects

All MSM individuals were recruited from the National Center for Global Health and Medicine. The study was approved by the ethics committee of Kumamoto University (RINRI-2289). Written informed consent was obtained from all individuals for the collection of peripheral blood and subsequent analysis according to the Declaration of Helsinki. An HIV-negative men who have sex with men (MSM) cohort was established at a sexual health clinic, the National Center for Global Health and Medicine in Tokyo, Japan. The HIV-1 status of MSM individuals was confirmed by a serological and p24 antigen enzyme Immunoassay (EIA) test every 3 months. Inclusion criteria for the selection of HESN individuals in the MSM cohort included (1) being able to speak and read Japanese, (2) being at least 16 years old, and (3) reporting anal sex (insertive or receptive) with a man. All had 10 or more points in the HIV Risk Index for MSM (HIRI-MSM), which includes questions regarding risk factors of HIV-1 infection, such as an experience of sex and/or anal sex with men and the presence of HIV-positive male sex partners.^63^ All of candidates were men in this study because the target of this study was MSM individuals. Therefore, gender was limited to men. Eligible men provided written consent, including allowing study staff to use their blood samples in our proposed study.

Six mL of peripheral blood from each individual was collected and used for this study. Approximately half of the individuals received PrEP within 12 months. PBMCs from these individuals were collected before PrEP was started.

#### Cell lines

The HLA-class Ia deficient 721.221 cell lines expressing CD4 and transfected with HLA-B∗51:01 (.221-B5101) and A∗02:06 (.221-A0206) were generated in a previous study.^64^^,^^65^ The cell lines were cultured in RPMI 1640 medium (Thermo Fisher Scientific, Waltham, MA, USA) containing 10% fetal bovine serum (FCS) and 0.15 mg/mL of hygromycin B (Merck Millipore, Burlington, MA, USA). C1R and 721.221 cells expressing other HLAs (see Figure 1) used in this study were generated previously^58^^,^^64^^,^^65^^,^^66^^,^^67^^,^^68^^,^^69^^,^^70^^,^^71^^,^^72^^,^^73^^,^^74^^,^^75^^,^^76^^,^^77^^,^^78^^,^^79^^,^^80^^,^^81^ and maintained in RPMI 1640 medium containing 10% FCS and 0.15 mg/mL of hygromycin B or 0.2 mg/mL of neomycin.

### Method details

#### HLA genotyping

HLA-A, -B, and -C genotypes were identified by the Luminex microbead method (Luminex 100 System; Luminex), and are reported according to the nomenclature of The HLA Dictionary 200432 at the NPO HLA Laboratory (Kyoto, Japan).

#### Preparation of HIV-1-infected .221 cells

An infectious provirus HIV-1, pNL432, was described previously.^82^ .221-B5101 cells or .221-A0206 cells were infected with the NL4-3 clone for 3 days. Infected cells were determined by intracellular staining with FITC-conjugated anti-HIV-1 p24 mAb (KC-57; Beckman Coulter, Brea, CA, USA).

#### HIV-1-specific T cell expansion and intracellular cytokine staining (ICS) assay

3×10^5^ or 6×10^5^ PBMCs were stimulated with 1 μM concentrations of the corresponding peptides and then cultured for 3 weeks in RPMI 1640 medium (Thermo Fisher Scientific, Waltham, MA, USA) containing 10% FCS, 10 ng/mL of human rIL-2 (PeproTech, Cranbury, NJ, USA), x1 MEM Non-Essential Amino Acids Solution (Thermo Fisher Scientific, Waltham, MA, USA), and 1 mM sodium pyruvate solution (Thermo Fisher Scientific, Waltham, MA, USA). Following T cell expansion, HLA class I expressing .221 cells pre-pulsed with each HIV-1 peptide or infected with HIV-1 NL4-3 were added to a 96-well plate together with bulk-cultured T cells, and then incubated for 4 h at 37°C with 10 g/mL brefeldin A (Sigma-Aldrich, St. Louis, MO, USA). For the screening ICS assay using peptide cocktails, the cells were then stained with allophycocyanin (APC)-labeled anti-CD8 mAb (BioLegend, San Diego, CA, USA). For the ICS assay using single peptides and HIV-1 NL4-3, the cells were then stained with Pacific blue (PB)-labeled anti-CD3 monoclonal antibody (mAb) (BioLegend, San Diego, CA, USA), fluorescein isothiocyanate (FITC)-labeled anti-CD19 mAb (BioLegend, San Diego, CA, USA), allophycocyanin (APC)-labeled anti-CD8 mAb (BioLegend, San Diego, CA, USA), and a LIVE/DEAD Fixable Near-IR Dead Cell Stain Kit (Thermo Fisher Scientific, Waltham, MA, USA). These stained cells were subsequently fixed with CellFIX (BD Biosciences, La Jolla, CA, USA) and incubated in permeabilization buffer (0.1% saponin/5% FBS/phosphate-buffered saline (PBS). Thereafter, the cells were stained with phycoerythrin (PE)-labeled anti-gamma interferon (anti-IFNγ) mAb (BioLegend, San Diego, CA, USA). Staining data were acquired on a FACSCanto II (BD Biosciences, La Jolla, CA, USA) and analyzed using FlowJo 10.8.1 software. The relative frequency of IFNγ^+^CD8^+^ cells was calculated as the frequency of IFNγ^+^ CD8^+^ cells among CD8^+^ cells stimulated with HLA class I^+^ .221 cells pre-pulsed with peptides minus that of IFNγ^+^CD8^+^ cells among CD8^+^ cells stimulated with .221 cells without peptides. A >0.2% relative frequency of IFNγ^+^CD8^+^ cells was selected to indicate potential positive responses and cases that showed a high response to .221 cells pre-pulsed with cocktail peptide(s) as a non-specific response were excluded.

### Quantification and statistical analysis

The relative frequency of IFNγ^+^CD8^+^ cells was calculated as the frequency of IFNγ^+^ CD8^+^ cells among CD8^+^ cells stimulated with HLA class I^+^ .221 cells pre-pulsed with peptides minus that of IFNγ^+^CD8^+^ cells among CD8^+^ cells stimulated with .221 cells without peptides. A >0.2% relative frequency of IFNγ^+^CD8^+^ cells was selected to indicate potential positive responses and cases that showed a high response to .221 cells pre-pulsed with cocktail peptide(s) as a non-specific response were excluded. Graphs were created in GraphPad Prism 9.5.1. In Figure 4, data of bar graph are presented as mean ± SD.

### Additional resources

This study is not clinical trials.

## Data Availability

• All data produced in this study are included in the published article and its supplementary information, or are available from the lead contact upon request. • This paper does not report original code. • Any additional information required to reanalyze the data reported in this paper is available from the lead contact upon request.

## Supplemental information

Table S1. List of well-defined CD8^+^ T cell epitopes, related to Figure 1
