# Supplementary material for: HIV-1 protective epitope-specific CD8+ T cells in HIV-1-exposed seronegative individuals
Source: iScience. 2023 Sep 29;26(11):108089. doi: 10.1016/j.isci.2023.108089 (PMC10589889; doi:10.1016/j.isci.2023.108089)
Supplement: Table S1. List of well-defined CD8+ T cell epitopes, related to Figure 1 [file mmc1.pdf]

iScience, Volume *26*

Supplemental information

HIV-1 protective epitope-specific $CD8^+$ T cells

in HIV-1-exposed seronegative individuals

Takayuki Chikata, Hiroyuki Gatanaga, Hung The Nguyen, Daisuke Mizushima, Yu Zhang, Nozomi Kuse, Shinichi Oka, and Masafumi Takiguchi

Table S1. List of well-defined CD8+ T cell epitopes

Cocktail	HLA allele	Epitope Name	Sequences	Reference
**1**	A*02	Gag IV9	IILGLNKIV	28
**1**	A*02:01/A*02:06	Pol YI9	YTAFTIPSI	30
**1**	A*02:01	Pol YV9	YQYMDDLYV	30
**1**	A*02:01	Gag FS8	FLGKIWPS	28
**1**	A*02:01	Pol IV10*	IYQYMDDLYV	30
**1**	A*02:01	Pol VV11*	VIYQYMDDLYV	30
**1**	A*02:01	Pol VL9	VIYQYMDDL	30
**1**	A*02:01	Gag SL9	SLYNTVATL	25
**1**	A*02:06	Nef GL9	GALDLSHFL	21
**1**	A*02:06	Gag RI9	RQANFLGKI	28
**1**	A*02:06	Pol SV9*#	SQIYAGIKV	24
**1**	A*02:06	Gag AA9*#	ATLEEMMTA	24, 28
**1**	A*02:06	Pol GL9	GQETAYFLL	21
**1**	A*02:06	Pol GI9	GQVDCSPGI	21
**1**	A*02:07	Gag YL9*	YVDRFYKTL	28
**2**	A*11:01	Pol IK9	IIATDIQTK	30
**2**	A*11:01	Nef AK9*	AVDLSHFLK	15
**2**	A*11:01	Gag TR8	TLYCVHQR	8
**2**	A*11:01	Nef QK10*	QVPLRPMTYK	15
**2**	A*11:01	Gag AK11	ACQGVGGPGHK	8, 15, 28
**2**	A*11:01	Pol QIK9	QIIEQLIKK	6, 8, 15
**2**	A*11:01	Pol AK9	AIFQSSMTK	8, 30
**2**	A*11:01	Pol QK9*	QIYAGIKVK	8
**2**	A*11:01	Pol AK10	AVFIHNFKRK	6
**2**	A*11:01	Env SK9*	SVITQACPK	8
**2**	A*11:01	Pol IK10	IYQEPFKNLK	30
**3**	A*24:02	Env RI11*	RYLRDQQLLGI	4
**3**	A*24:02	Pol IL9#	IYQEPFKNL	30
**3**	A*24:02	Gag ML8	MYSPTSIL	28
**3**	A*24:02	Env RL9*	RYLRDQQLL	4
**3**	A*24:02	Env SL9	SYRRLRDLL	4
**3**	A*24:02	Env FF9*	FYCNTTQLF	4
**3**	A*24:02	Env WI9*	WYIKIFIMI	4
**3**	A*24:02	Gag KW9*	KYKLKHIVW	13
**3**	A*24:02	Env RL8*	RYLRDQQL	4
**3**	A*24:02	Gag IL10	IYKRWIILGL	4
**3**	A*24:02	Gag IL8	IYKRWIIL	4
**3**	A*24:02	Gag NL8	NYPIVQNL	1, 4
**3**	A*24:02	Nef RW8*	RYPLTFGW	23
**3**	A*24:02	Nef RF10*	RYPLTFGWCF	4,23
**3**	A*24:02	Gag DT9	DYVDRFYKT	28
**4**	A*26:01	Pol EY9*	ETKLGKAGY	10
**4**	A*26:01	Pol EY10	EVNIVTDSQY	10
**4**	A*26:01	Env EM10	EVFRPGGGDM	10
**4**	A*26:02	Gag TM9*	TLQEQIGWM	28
**4**	A*26:03	Env EA10*	EVHNVWATHA	30
**4**	A*26:01/A*26:03	Gag EL9*	EVIPMFSAL	10, 12
**4**	A*31:01	Nef KR9*	KLAFHHMAR	11

**4**	A*31:01	Gag IK10*	IAKNCRAPRK	28
**5**	A*33:03	Pol KR10	KIQNFRVYYR	9
**5**	A*33:03	Gag HR10*#	HIAKNCRAPR	28
**5**	A*33:03	Gag DR11	DYVDRFYKTLR	28
**5**	A*33:03	Gag MR9*	MVHQAISPR	9
**5**	A*33:03	Env VR10*	VFAVLSIVNR	7
**5**	A*33:03	Env VIR10*	VIEVAQRAYR	7
**5**	A*33:03	Pol FR9*	FYVDGAANR	9
**5**	A*33:03	Env ER8*	EVAQRAYR	7
**5**	A*33:03	Pol TR11*	TLWQRPLVTIR	9
**5**	A*33:03	Pol ER10*#	ELKKIIGQVR	30, 21
**6**	B*07:02	Pol SM9#	SPAIFQSSM	30
**6**	B*07:02	Gag GL9	GPGHKARVL	28
**6**	B*15:01	Nef TY11	TQGYFPDWQNY	24
**6**	B*15:01	Gag GY9*	GLNKIVRMY	28
**6**	B*15:01	Pol IQY11*	IQKQGQGQWTY	30
**6**	B*39:01	Pol EA10*	EHLKTAVQMA	30
**6**	B*39:01	Pol TL9*	THLEGKIIL	30
**7**	B*35:01	Pol VY10*	VPLDKDFRKY	29, 30
**7**	B*35:01	Nef RY11*	RPQVPLRPMTY	29
**7**	B*35:01	Nef FL9*	FPVRPQVPL	3
**7**	B*35:01	Env RL9	RPIVSTQLL	2, 3
**7**	B*35:01	Env DL9*	DPNPQEVVL	29
**7**	B*35:01	Gag NY10	NPPIPVGEIY	29
**7**	B*35:01	Pol IY11	IPAETGQETAY	29
**7**	B*35:01	Pol SM9	SPAIFQSSM	2, 3
**7**	B*35:01	Nef YF9*	YPLTFGWCF	29
**7**	B*35:01	Pol TY9*	TVLDVGDAY	29, 30
**7**	B*35:01	Pol NQY9*	NPDIVIYQY	29, 30
**7**	B*35:01	Nef VY8*	VPLRPMTY	29
**7**	B*35:01	Pol EY10*	EPIVGAETFY	29
**7**	B*35:01	Gag HA9*	HPVHAGPIA	29
**7**	B*35:01	Env TVW9	TVYYGVPVW	29
**7**	B*35:01	Env TW9	TAVPWNAS	29
**7**	B*35:01	Gag NY9	NSSQVSQNY	29
**7**	B*35:01	Rev KY10	KTVRLIKFLY	29
**7**	B*35:01	Pol IL9	IPLTEEAEL	2, 3
**7**	B*35:01	Env VL11	VPVWKEATTTL	29
**8**	B*40:02	Gag TL8*#	TERQANFL	28
**8**	B*40:02	Gag EG11	EWDRLHPVHAG	21
**8**	B*40:02	Pol GI8*#	GERIVDII	24, 30
**8**	B*40:02	Pol TL8	TDIQTKEL	17, 24
**8**	B*40:02	Gag KL9	KELYPLASL	24
**8**	B*40:02	Pol EL11	ETGQETAYFLL	17, 22
**8**	B*40:02	Gag AP8	AEWDRLHP	21
**8**	B*40:02	Gag RV8	RELERFAV	21
**8**	B*40:06	Pol GA9*#	GERIVDIIA	30
**8**	B*40:06	Pol IT10*#	IEAEVIPAET	24
**8**	B*40:06	Pol LA9*#	LEGKIILVA	24, 30
**9**	B*51:01/B*52:01	Pol TI8*#	TAFTIPSI	5, 30

**9**	B*51:01	Env LI9*	LPCRIKQII	5
**9**	B*51:01	Gag NI9*	NANPDCKTI	5
**9**	B*51:01	Rev VL11*	VPLQLPPLERL	16
**9**	B*51:01	Env RI9*	RAYRAILHI	5
**9**	B*51:01	Pol LI9**	LPPVVAKEI	5
**9**	B*51:01	Pol DL8*	DAYFSVPL	5
**9**	B*51:01	Gag YI9*	YAPPIGGQI	5
**9**	B*51:01	Pol QI9*	QGWKGSPAI	5
**9**	B*52:01	Gag MI8*#	MQMLKETI	24
**9**	B*52:01	Pol LI8*	LQKQITKI	26
**9**	B*52:01	Pol WI8	WQRPLVTI	26
**9**	B*52:01	Gag WV8*#	WMTETLLV	24, 28
**9**	B*52:01	Gag RI8*#	RMYSPTSI	24, 28
**9**	B*52:01	Pol SI8*#	SQYALGII	24
**10**	B*48:01	Gag VL9*	VKNWMTETL	14
**10**	B*48:01	Gag GI8*	GQMVHQAI	19
**10**	B*48:01	Gag LI10	LQGQMVHQAI	19
**10**	B*48:01	Gag RI9*	RQANFLGKI	14
**10**	B*54:01	Pol FT8	FPISPIET	20
**10**	B*54:01	Pol FP10*	FPISPIETVP	20
**10**	B*54:01	Pol FV11*	FPISPIETVPV	20
**10**	B*54:01	Pol FV9*	FPISPIETV	20
**10**	B*67:01	Gag NL11*#	NPDCKTILKAL	24, 28
**10**	B*67:01	Gag TL9*#	TPQDLNTML	24
**10**	B*67:01	Nef RM9*	RPQVPLRPM	33
**10**	B*67:01	Nef RL9*	RPMTYKGAL	33
**10**	B*67:01	Pol PL11*	PLVTIKIGGQL	33
**10**	B*67:01	Pol AL10*	AQPDKSESEL	33
**10**	B*67:01	Nef YF9	YPLTFGWCF	33
**11**	C*03	Gag KL9	KALGPAATL	28
**11**	C*03	Gag YL9	YVDRFYKTL	28
**11**	C*08	Gag KL9*	KALGPAATL	28
**11**	C*08:01	Pol IL8*	IYQYMDDL	30
**11**	C*14:02/C*14:03	Gag AM9 *	AFSPEVIPM	32
**11**	C*14:02/C*14:03	Gag LY9	LYNTIAVLY	32
**11**	C*14:02/C*14:03	Nef YT9*	YFPDWQNYT	32
**11**	C*14:02/C*14:03	Nef IF8*	IYHTQGYF	32
**11**	C*14:02/C*14:03	Nef AL9	AFHHVAREL	32
**11**	C*14:02/C*14:03	Pol KM9	KYHSNWRAM	32
**12**	C*01:02	Gag YI9	YSPTSILDI	28
**12**	C*12:02	Pol KY9	KQNPDIVIY	18
**12**	C*12:02	Pol IY11*#	ILKEPVHGVYY	26, 27
**12**	C*12:02	Nef MY9*#	MARELHPEY	27
**12**	C*12:02	Gag TH9	TVATLYCVH	27
**12**	C*12:02	Env RL9*	RAIEAQQHL	31

* Immunodominat epitope; the frequency of responders among Japanese HIV1-infected individuals who carry correspondant HLA allele was >20%

Protective epitope

Supplemental references

1. Fujiwara, M., Tanuma, J., Koizumi, H., Kawashima, Y., Honda, K., Mastuoka-Aizawa, S., Dohki, S., Oka, S., and Takiguchi, M. (2008). Different abilities of escape mutant-specific cytotoxic T cells to suppress replication of escape mutant and wild-type human immunodeficiency virus type 1 in new hosts. J Virol *82*, 138-147. 10.1128/JVI.01452-07.
2. Shiga, H., Shioda, T., Tomiyama, H., Takamiya, Y., Oka, S., Kimura, S., Yamaguchi, Y., Gojoubori, T., Rammensee, H.G., Miwa, K., and Takiguchi, M. (1996). Identification of multiple HIV-1 cytotoxic T-cell epitopes presented by human leukocyte antigen B35 molecules. AIDS *10*, 1075-1083.
3. Tomiyama, H., Miwa, K., Shiga, H., Moore, Y.I., Oka, S., Iwamoto, A., Kaneko, Y., and Takiguchi, M. (1997). Evidence of presentation of multiple HIV-1 cytotoxic T lymphocyte epitopes by HLA-B*3501 molecules that are associated with the accelerated progression of AIDS. J Immunol *158*, 5026-5034.
4. Ikeda-Moore, Y., Tomiyama, H., Miwa, K., Oka, S., Iwamoto, A., Kaneko, Y., and Takiguchi, M. (1997). Identification and characterization of multiple HLA-A24-restricted HIV-1 CTL epitopes: strong epitopes are derived from V regions of HIV-1. J Immunol *159*, 6242-6252.
5. Tomiyama, H., Sakaguchi, T., Miwa, K., Oka, S., Iwamoto, A., Kaneko, Y., and Takiguchi, M. (1999). Identification of multiple HIV-1 CTL epitopes presented by HLA-B*5101 molecules. Hum Immunol *60*, 177-186. 10.1016/s0198-8859(98)00113-x.
6. Fukada, K., Chujoh, Y., Tomiyama, H., Miwa, K., Kaneko, Y., Oka, S., and Takiguchi, M. (1999). HLA-A*1101-restricted cytotoxic T lymphocyte recognition of HIV-1 Pol protein. AIDS *13*, 1413-1414. 10.1097/00002030-199907300-00021.
7. Hossain, M.S., Tomiyama, H., Inagawa, T., Sriwanthana, B., Oka, S., and Takiguchi, M. (2001). HLA-A*3303-restricted cytotoxic T lymphocyte recognition for novel epitopes derived from the highly variable region of the HIV-1 Env protein. AIDS *15*, 2199-2201. 10.1097/00002030-200111090-00020.
8. Fukada, K., Tomiyama, H., Wasi, C., Matsuda, T., Kusagawa, S., Sato, H., Oka, S., Takebe, Y., and Takiguchi, M. (2002). Cytotoxic T-cell recognition of HIV-1 cross-clade and clade-specific epitopes in HIV-1-infected Thai and Japanese patients. AIDS *16*, 701-711. 10.1097/00002030-200203290-00005.
9. Hossain, M.S., Tomiyama, H., Inagawa, T., Ida, S., Oka, S., and Takiguchi, M. (2003). Identification and characterization of HLA-A*3303-restricted, HIV type 1 Pol- and Gag-derived cytotoxic T cell epitopes. AIDS Res Hum Retroviruses *19*, 503-510. 10.1089/088922203766774559.
10. Satoh, M., Takamiya, Y., Oka, S., Tokunaga, K., and Takiguchi, M. (2005). Identification and characterization of HIV-1-specific CD8+ T cell epitopes presented by HLA-A*2601. Vaccine *23*, 3783-3790. 10.1016/j.vaccine.2005.02.022.
11. Borghan, M.A., Oka, S., and Takiguchi, M. (2005). Identification of HLA-A*3101-restricted cytotoxic T-lymphocyte response to human immunodeficiency virus type 1 (HIV-1) in patients with chronic HIV-1 infection. Tissue Antigens *66*, 305-313.

10.1111/j.1399-0039.2005.00489.x.
12. Kawashima, Y., Satoh, M., Oka, S., Shirasaka, T., and Takiguchi, M. (2008). Different immunodominance of HIV-1-specific CTL epitopes among three subtypes of HLA-A*26 associated with slow progression to AIDS. Biochem Biophys Res Commun *366*, 612-616. 10.1016/j.bbrc.2007.11.031.
13. Koizumi, H., Iwatani, T., Tanuma, J., Fujiwara, M., Izumi, T., Oka, S., and Takiguchi, M. (2009). Escape mutation selected by Gag28-36-specific cytotoxic T cells in HLA-A*2402-positive HIV-1-infected donors. Microbes Infect *11*, 198-204. 10.1016/j.micinf.2008.11.005.
14. Murakoshi, H., Kitano, M., Akahoshi, T., Kawashima, Y., Dohki, S., Oka, S., and Takiguchi, M. (2009). Identification and characterization of 2 HIV-1 Gag immunodominant epitopes restricted by Asian HLA allele HLA-B*4801. Hum Immunol *70*, 170-174. 10.1016/j.humimm.2008.12.011.
15. Koizumi, H., Hashimoto, M., Fujiwara, M., Murakoshi, H., Chikata, T., Borghan, M.A., Hachiya, A., Kawashima, Y., Takata, H., Ueno, T., et al. (2010). Different in vivo effects of HIV-1 immunodominant epitope-specific cytotoxic T lymphocytes on selection of escape mutant viruses. J Virol *84*, 5508-5519. 10.1128/JVI.02483-09.
16. Kawashima, Y., Kuse, N., Gatanaga, H., Naruto, T., Fujiwara, M., Dohki, S., Akahoshi, T., Maenaka, K., Goulder, P., Oka, S., and Takiguchi, M. (2010). Long-term control of HIV-1 in hemophiliacs carrying slow-progressing allele HLA-B*5101. J Virol *84*, 7151-7160. 10.1128/JVI.00171-10.
17. Watanabe, T., Murakoshi, H., Gatanaga, H., Koyanagi, M., Oka, S., and Takiguchi, M. (2011). Effective recognition of HIV-1-infected cells by HIV-1 integrase-specific HLA-B *4002-restricted T cells. Microbes Infect *13*, 160-166. 10.1016/j.micinf.2010.10.006.
18. Honda, K., Zheng, N., Murakoshi, H., Hashimoto, M., Sakai, K., Borghan, M.A., Chikata, T., Koyanagi, M., Tamura, Y., Gatanaga, H., et al. (2011). Selection of escape mutant by HLA-C-restricted HIV-1 Pol-specific cytotoxic T lymphocytes carrying strong ability to suppress HIV-1 replication. Eur J Immunol *41*, 97-106. 10.1002/eji.201040841.
19. Naruto, T., Murakoshi, H., Chikata, T., Koyanagi, M., Kawashima, Y., Gatanaga, H., Oka, S., and Takiguchi, M. (2011). Selection of HLA-B57-associated Gag A146P mutant by HLA-B *48:01-restricted Gag140-147-specific CTLs in chronically HIV-1-infected Japanese. Microbes Infect *13*, 766-770. 10.1016/j.micinf.2011.03.009.
20. Hashimoto, M., Akahoshi, T., Murakoshi, H., Ishizuka, N., Oka, S., and Takiguchi, M. (2012). CTL recognition of HIV-1-infected cells via cross-recognition of multiple overlapping peptides from a single 11-mer Pol sequence. Eur J Immunol *42*, 2621-2631. 10.1002/eji.201242483.
21. Watanabe, K., Murakoshi, H., Tamura, Y., Koyanagi, M., Chikata, T., Gatanaga, H., Oka, S., and Takiguchi, M. (2013). Identification of cross-clade CTL epitopes in HIV-1 clade A/E-infected individuals by using the clade B overlapping peptides. Microbes Infect *15*, 874-886. 10.1016/j.micinf.2013.08.002.
22. Rahman, M.A., Kuse, N., Murakoshi, H., Chikata, T., Gatanaga, H., Oka, S., and Takiguchi, M. (2014). Raltegravir and elvitegravir-resistance mutation E92Q affects

HLA-B*40:02-restricted HIV-1-specific CTL recognition. Microbes Infect *16*, 434-438. 10.1016/j.micinf.2014.03.003.
23. Sun, X., Fujiwara, M., Shi, Y., Kuse, N., Gatanaga, H., Appay, V., Gao, G.F., Oka, S., and Takiguchi, M. (2014). Superimposed epitopes restricted by the same HLA molecule drive distinct HIV-specific CD8+ T cell repertoires. J Immunol *193*, 77-84. 10.4049/jimmunol.1400375.
24. Murakoshi, H., Akahoshi, T., Koyanagi, M., Chikata, T., Naruto, T., Maruyama, R., Tamura, Y., Ishizuka, N., Gatanaga, H., Oka, S., and Takiguchi, M. (2015). Clinical Control of HIV-1 by Cytotoxic T Cells Specific for Multiple Conserved Epitopes. J Virol *89*, 5330-5339. 10.1128/JVI.00020-15.
25. Lissina, A., Chakrabarti, L.A., Takiguchi, M., and Appay, V. (2016). TCR clonotypes: molecular determinants of T-cell efficacy against HIV. Curr Opin Virol *16*, 77-85. 10.1016/j.coviro.2016.01.017.
26. Murakoshi, H., Koyanagi, M., Chikata, T., Rahman, M.A., Kuse, N., Sakai, K., Gatanaga, H., Oka, S., and Takiguchi, M. (2017). Accumulation of Pol Mutations Selected by HLA-B*52:01-C*12:02 Protective Haplotype-Restricted Cytotoxic T Lymphocytes Causes Low Plasma Viral Load Due to Low Viral Fitness of Mutant Viruses. J Virol *91*. 10.1128/JVI.02082-16.
27. Chikata, T., Murakoshi, H., Koyanagi, M., Honda, K., Gatanaga, H., Oka, S., and Takiguchi, M. (2017). Control of HIV-1 by an HLA-B*52:01-C*12:02 Protective Haplotype. J Infect Dis *216*, 1415-1424. 10.1093/infdis/jix483.
28. Murakoshi, H., Zou, C., Kuse, N., Akahoshi, T., Chikata, T., Gatanaga, H., Oka, S., Hanke, T., and Takiguchi, M. (2018). CD8(+) T cells specific for conserved, cross-reactive Gag epitopes with strong ability to suppress HIV-1 replication. Retrovirology *15*, 46. 10.1186/s12977-018-0429-y.
29. Murakoshi, H., Koyanagi, M., Akahoshi, T., Chikata, T., Kuse, N., Gatanaga, H., Rowland-Jones, S.L., Oka, S., and Takiguchi, M. (2018). Impact of a single HLA-A*24:02-associated escape mutation on the detrimental effect of HLA-B*35:01 in HIV-1 control. EBioMedicine *36*, 103-112. 10.1016/j.ebiom.2018.09.022.
30. Zou, C., Murakoshi, H., Kuse, N., Akahoshi, T., Chikata, T., Gatanaga, H., Oka, S., Hanke, T., and Takiguchi, M. (2019). Effective Suppression of HIV-1 Replication by Cytotoxic T Lymphocytes Specific for Pol Epitopes in Conserved Mosaic Vaccine Immunogens. J Virol *93*. 10.1128/JVI.02142-18.
31. Chikata, T., Paes, W., Akahoshi, T., Partridge, T., Murakoshi, H., Gatanaga, H., Ternette, N., Oka, S., Borrow, P., and Takiguchi, M. (2019). Identification of Immunodominant HIV-1 Epitopes Presented by HLA-C*12:02, a Protective Allele, Using an Immunopeptidomics Approach. J Virol *93*. 10.1128/JVI.00634-19.
32. Chikata, T., Paes, W., Kuse, N., Partridge, T., Gatanaga, H., Zhang, Y., Kuroki, K., Maenaka, K., Ternette, N., Oka, S., et al. (2022). Impact of Micropolymorphism Outside the Peptide Binding Groove in the Clinically Relevant Allele HLA-C*14 on T Cell Responses in HIV-1 Infection. J Virol *96*, e0043222. 10.1128/jvi.00432-22.
33. Zhang, Y., Chikata, T., Kuse, N., Murakoshi, H., Gatanaga, H., Oka, S., and Takiguchi, M. (2022). Immunological Control of HIV-1 Disease Progression by Rare Protective HLA Allele. J Virol *96*, e0124822. 10.1128/jvi.01248-22.
